# The use of whole body diffusion-weighted post-mortem magnetic resonance imaging in timing of perinatal deaths

**DOI:** 10.1007/s00414-018-1906-5

**Published:** 2018-07-28

**Authors:** Susan C. Shelmerdine, Cheryl Main, John Ciaran Hutchinson, Dean Langan, Neil J. Sebire, Owen J. Arthurs

**Affiliations:** 1 Great Ormond Street Hospital for Children NHS Foundation Trust, London, WC1N 3JH UK; 2 UCL Great Ormond Street Institute of Child Health, 30 Guilford Road, London, WC1N 1EH UK; 3 University Hospital Southampton NHS Foundation Trust, Southampton, SO16 6YD UK

**Keywords:** Autopsy, Post-mortem, MRI, Diffusion, Perinatal, Paediatric

## Abstract

**Objectives:**

Diffusion-weighted MRI provides information regarding body water movement following death, which may be an imaging marker of post-mortem interval (time since death; PMI) or maceration (degree of tissue degradation during intra-uterine retention) in perinatal deaths. Our aim was to evaluate the relationship between maceration, PMI and body organ apparent diffusion coefficient (ADC) values in a cohort of subjects across a wide gestational range.

**Materials:**

Whole body post-mortem MRI with diffusion-weighted imaging (DWI) sequences were performed at 1.5 T, with *b* values of 0, 500 and 1000 mm^2^/s. Mean ADC values were calculated from regions of interest (ROIs) placed in the lungs, myocardium, spleen, renal cortex, liver and psoas muscle by two independent readers. Multivariable regression analysis was performed against PMI, gestational age, post-mortem weight, maceration score and gender.

**Results:**

Eighty perinatal deaths were imaged with mean gestational age of 32 weeks (18–41 weeks), of which 49 (61.3%) were male. The mean PMI was 8 days (1–18 days). Maceration scores were statistically significant predictive factors for ADC values in all included body organs except the lungs, but PMI was not a predictor for ADC values in any body organ. In the absence of maceration (*n* = 14), PMI was not statistically associated with ADC values in any of the body areas. The ratio of agreement in the majority of body areas was close to 1 (range between 0.95 and 1.10).

**Conclusion:**

Maceration, not PMI, is significantly associated with ADC values in perinatal deaths. Further research is needed to understand organ-specific changes in the post-mortem period.

## Introduction

Post-mortem magnetic resonance imaging (PMMR) has been shown to have a high diagnostic accuracy in identification of perinatal abnormalities and cause of death [1, 2]. Diffusion-weighted imaging (DWI), a sequence that is used in PMMR imaging protocols [3], derives contrast from the differences in movement of water molecules within various tissues [4] and can be indirectly quantified using the apparent diffusion coefficient (ADC) [5]. This value can provide information relating to the microscopic structure and functional changes at a cellular level within body organs where structures with high cellularity (i.e. causing restriction of movement of water molecules) demonstrate low ADC values, compared to less cellular structures with higher ADC values [6]. In live children, this imaging sequence has shown to have promising clinical uses, such as for the assessment of tumour response to treatment [7] and in the identification of inflammatory changes (such as in inflammatory bowel disease [8] and enthesitis-related arthritis [9]).

**Fig. 1:**
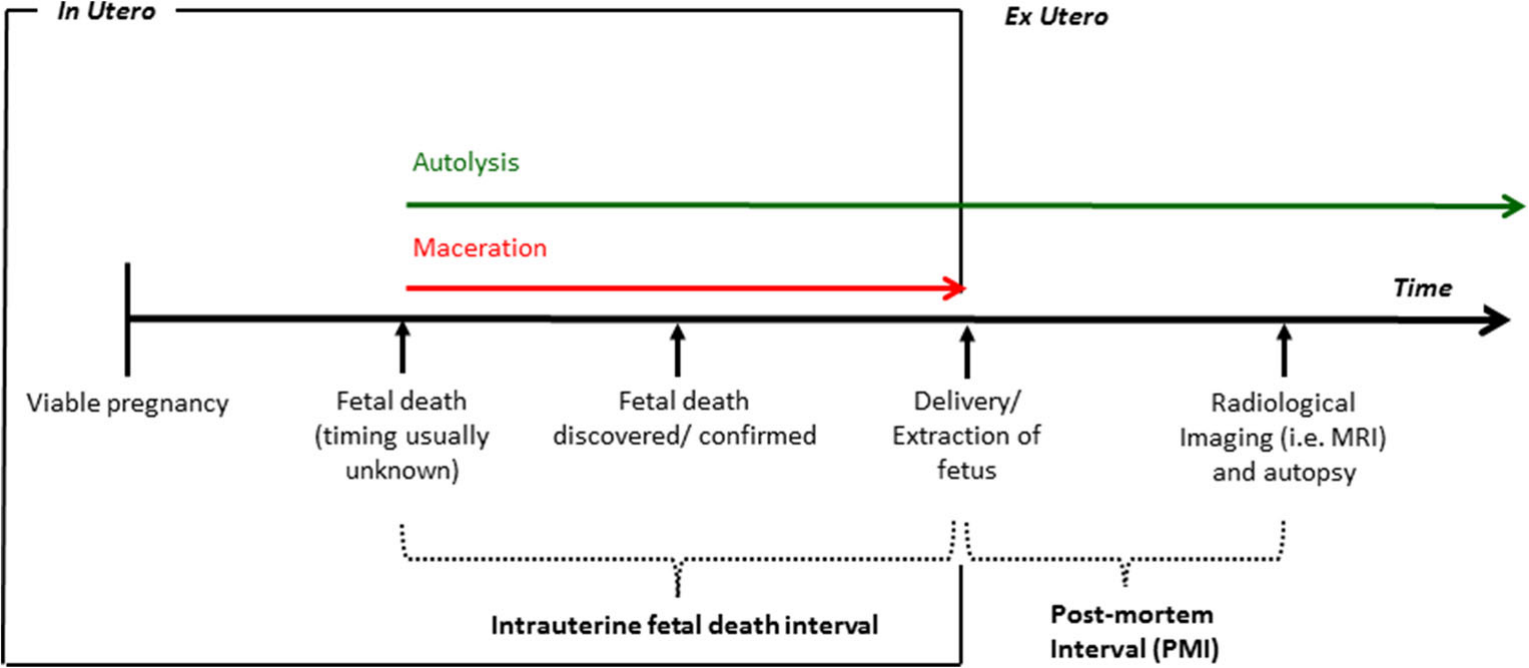
Illustration of the timing of events where intra-uterine foetal demise has occurred, to highlight the terms ‘post-mortem interval (PMI)’ and intra-uterine death interval. The degradation of tissue via autolysis (green arrow) occurs after the death of the foetus, whereas those of maceration (red arrow) occur only whilst the foetus remains in utero. In cases where the foetus is born alive and subsequently dies, maceration by definition will not have occurred and the body will only undergo autolysis

The application of DWI in the post-mortem setting is appealing as it (a) does not require the administration of an exogenous contrast agent, (b) the irreversible and progressive cellular changes that occur as a consequence of autolysis should be detectable and (c) could result in ADC values that change predictably over time, and may allow the estimation of time since death, or post-mortem interval (PMI). Nevertheless, imaging assessment of perinatal post-mortem tissue has several confounders. In addition to autolysis (i.e. the enzymatic degradation of tissue following death), foetuses which have suffered intra-uterine demise and retention prior to delivery undergo a process of maceration (i.e. tissue degeneration within a fluid-filled cavity (amniotic fluid)). Figure 1 provides a diagrammatical representation of the relative timing of these processes. Given that exact knowledge of an intra-uterine death is frequently unknown (and thus the timing of intra-uterine retention inaccurate), the degree of maceration at autopsy by external assessment is commonly used as a subjective, surrogate marker to estimate intra-uterine retention time [10].

Reports of PMMR imaging in the medical literature which have used DWI have shown that in the foetal brain (specifically the basal ganglia) [11] and lung [12] the ADC value changes are associated with PMI. Maceration was also found to affect ADC values in most areas of the foetal brain, but other body organs have not been comprehensively evaluated.

The aim of this study was therefore to evaluate the relationship between maceration, PMI and body organ ADC values across a wide gestational age range in the setting of perinatal death. If the ADC values in body organs are found to be robust marker of PMI or maceration, this could have implications for the investigation of intra-uterine foetal deaths, especially where timing may be medico-legally important.

## Methods

### Study cohort

Written informed consent was obtained from all parents of participants for clinical pre-autopsy PMMR, as part of our institution’s post-mortem imaging protocol. DWI sequences were prospectively performed on all post-mortem foetal, stillbirth and early neonatal deaths referred to our institution from May 2013 to September 2016 (40 months). Cases in which the DWI imaging was of inadequate quality, incomplete, or where there was significant organ abnormality on imaging or at autopsy were excluded. Bodies were stored in the mortuary at 4 °C within 24 h of delivery and PMMR was performed during dedicated research imaging lists to prevent any disruption to usual clinical services.

Demographic included age at time of death (for neonatal deaths, in days), gestational age (in weeks) and post-mortem interval (PMI, days from delivery to imaging). Intra-uterine retention interval (time between foetal death and delivery/extraction) was not recorded due to unreliable timing and inconsistent documentation in the notes. For this reason, we used maceration score (scored subjectively by the duty pathologist: 0–none, 1–mild or moderate, 2–established [10]).

### Magnetic resonance imaging

All MR imaging was performed at 1.5 T (Avanto, Siemens Medical Solutions, Erlangen, Germany) with a conventional phase array body coil. Body imaging included isovolumetric T1- and T2-weighted imaging for the whole body (neck to feet) with additional isovolumetric T2-weighted constructive interface stead state (CISS) sequences covering the thorax (for cardiac anomalies) and a 2D axial T2-weighted inversion recovery sequence of the body, according to published protocols [3]. The coil used was dependent on patient size, selected to allow for best image quality.

Diffusion-weighted imaging sequences were performed using single-shot spin echo-planar imaging (EPI) in the axial plane, with the following parameters: 19 slices in three non-collinear axis directions, EPI factor 95, TR 2700 ms; TE 96 ms; FOV 230 mm; 128 × 128 matrix, 5-mm-slice thickness with 1-mm gap, acquisition time 90 s. Diffusion gradient values were *b* = 0, 500 and 1000 s/mm^2^.

### DWI analysis

From the native DWI acquisition, ADC maps were obtained on the acquisition console (Syngo, Siemens Medical Solutions, Erlangen, Germany). Data was then transferred to Osirix (open source DiCOM viewer: http://www.osirix-viewer.com) for ADC measurements in different regions of interest (ROI). Circular ROIs were drawn manually and plotted on ADC maps for all cases by two independent readers blinded to one another’s findings and also to the post-mortem report. Where there was difficulty in identifying the delineation of the target organ, cross-referencing was made to the isovolumetric T2 sequences obtained during the same MRI examination for anatomical information. The readers read the cases once. Both readers were qualified paediatric radiologists with 7 years experience each.

**Fig. 2:**
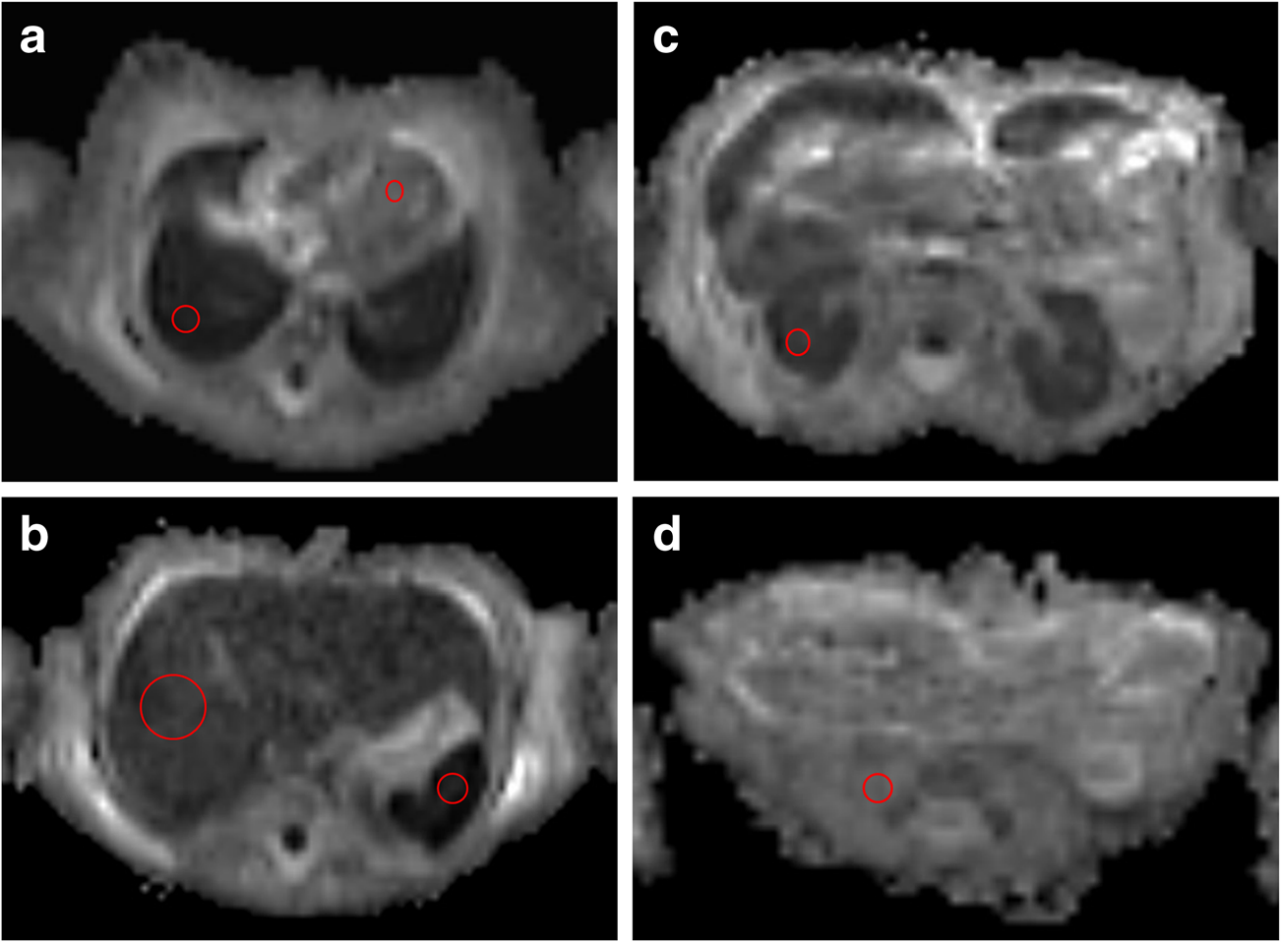
Foetal thoraco-abdominal post-mortem axial apparent diffusion coefficient (ADC) maps from DWI sequences (**a**–**d**) in a 32 gestational age foetus. The axial slices illustrate the lungs and myocardium (**a**), liver and spleen (**b**), kidneys (**c**) and psoas muscle (**d**) with corresponding regions of interest drawn

The ROIs were placed on the following body locations (Fig. 2):

1. Lung: obtained within the largest region of ‘normal’ lung parenchyma (i.e. non-consolidated regions for neonatal deaths, and for all cases—not including any pleural effusions).
2. Myocardium: within the intraventricular septum or left ventricular muscle, whichever allowed the largest ROI volume to be obtained.
3. Right renal cortex: avoiding any renal pelvis.
4. Liver: within the right lobe of the liver to obtain the most representative ROI sample.
5. Spleen: within the centre of the organ to include the largest ROI possible
6. Right psoas muscle: used as a control value.

For each ROI, a mean ADC value (× 10^−5^ mm^2^/s) ± standard deviation (SD) was obtained. The circular area for the ROI was drawn to include as much of the target organ as possible, whilst maintaining a relative SD < 10% of the mean to ensure homogeneity, typically < 25mm^2^. Size-specific ROIs were used for each individual case rather than choosing a fixed ROI, due to the range of foetal sizes (weight and gestation range given in Table 1) in our sample size. Where a valid ADC value for an organ could not be obtained (either due to a small organ inclusion area on the ADC map or significant artefact), then this was left blank or unrecorded.

**Table 1:** Demographic data of the 80 perinatal deaths included in this study. The neonatal sub-group (*n* = 14) were delivered alive but died within the first month of life.

	Case group	*N*	Mean	Median	IQR	Range
Age at death (days)	Neonatal deaths	14	7	1	0, 14.8	0–25
Gestational age (weeks)	Neonatal deaths	14	34	37	33, 40	22–40
Gestational age (weeks)	Stillbirths, miscarriages and terminations*	66	32	34	27, 38	18–41
Post-mortem weight (kg)	All cases	80	2.06	2.292	0.783, 3.006	0.258–4.73
Post-mortem interval (days)	All cases	80	8	7.5	5, 10	1–18

*The remainder of our cases which were not neonatal deaths were born without signs of life. Their date of death and date of birth are the same and age at death is therefore 0 days

Temperature correction for ADC values was not applied given that our sample included only post-mortem cases, imaged and stored at comparable temperatures. No comparison or extrapolation of our values with live cases was performed.

### Statistical analysis

Multilevel models were fitted for each ROI with mean corrected ADC values (10^−5^ mm^2^/s) with the outcome variable subject and reader both included as complete cross-classified random intercepts. Fixed effect predictor variables include maceration scores (MAC), post-mortem interval (PMI), gestational age, gender and post-mortem weight. Given a prior publication stating maceration to be a strongest predictor in ADC values for post-mortem foetal brain [11], we extracted a sub-group of cases in whom maceration could not have occurred (i.e. neonatal deaths; babies who were born alive and subsequently died). The mean corrected ADC values for each ROI were plotted against the post-mortem interval and the adjusted *R*^2^ estimates were calculated to summarise the strength of any linear relationships for the various body organs. This subsample contained 14 patients, and therefore given the low sample size, it was not possible to adjust for multiple predictors.

To measure inter-reader variability, Bland-Altman plots were constructed with 95% limits of agreement on the ratio between the two reader mean ADC values. The ratios of the two measurements were calculated, instead of the absolute difference, because agreement is dependent on the scale of the ADC measurement. All analyses were carried out in R version 3.3.0 and the R package *lme4* version 1.1.141 was used for multilevel analysis.

## Results

### Study sample

We identified 80 (49, 61.3% male) perinatal deaths for inclusion in the study: 14 (17.5%) neonatal deaths, 21(26.2%) termination of pregnancies (TOP), 2 (2.5%) miscarriages and 43 (53.8%) stillbirth/intra-uterine deaths. The patient demographics are given in Table 1. Table 2 lists the mean ADC values for the different body areas based on the total number of readings obtained by both readers.

**Table 2:** Summary of body organ ADC measurements (×10^−5^ mm^2^/s) in all 80 perinatal death cases, by both readers. The *N* numbers states the number of observations that the mean and range ADC values are derived from (i.e. if both readers could obtain an ADC score for all cases, then *N* = 160)

	*N* (total no. observations)	Median	Mean	Range	IQR	SD
Lung ADC	145	431	448	171–1073	375, 514	45
Myocardium ADC	136	610	615	224–1231	399, 783	58
Right renal cortex ADC	160	422	454	195–1046	315, 531	48
Liver ADC	159	450	439	66–829	279, 581	50
Spleen ADC	148	276	329	66–857	221, 386	64
Right psoas ADC	159	585	594	352–1069	486, 687	65

### Multivariate analysis

Using multilevel modelling, six prediction models were fitted for the lungs, myocardium, renal cortex, liver, spleen and psoas muscle. Maceration score was a statistically significant predictor for ADC values in all body areas except the lungs (Table 3). The other five models predict an increase in ADC values of between 93.82 (spleen, 95% CI 31.19–156.4) and 321.8 (myocardium, 95% CI 230.1–413.5) in patients with a maceration score of 2 (in comparison with maceration score 0). Post-mortem interval (PMI) was not a predictor for ADC values for any of the body organs after adjusting for all other predictor variables.

**Table 3:** Stepwise multivariate linear models. Multivariate linear regression, using potential predictors of reader, maceration score, gestational age, gender, postmortem (PM) weight and postmortem interval (PMI) in each model. Statistically significant results are highlighted in bold and in italics

Dependent variable	Independent variables	*B*	s.e.	95% CI for B		*p* value
				Lower	Upper	
Lung	(Constant)	417.2	128.1	–	–	–
	Maceration score 1 (ref = 0)	30.04	42.76	− 53.78	113.9	0.485
	Maceration score 2 (ref = 0)	11.13	30.86	− 49.36	71.61	0.720
	Gestational age (weeks)	− 1.363	4.996	− 11.16	8.429	0.786
	Gender (ref = female)	− 3.037	26.64	− 55.25	49.18	0.910
	PM weight (kg)	6.523	25.67	− 43.79	56.84	0.800
	PMI	6.949	4.138	− 1.162	15.06	0.098
Myocardium	(Constant)	400.2	193.2	–	–	–
	*Maceration score 1 (ref = 0)*	*247.6*	*65.97*	*118.3*	*376.9*	***< 0.001***
	*Maceration score 2 (ref = 0)*	*321.8*	*46.79*	*230.1*	*413.5*	***< 0.001***
	Gestational age (weeks)	3.674	7.536	− 11.1	18.44	0.628
	Gender (ref = female)	0.618	40.91	− 79.56	80.8	0.988
	PM weight (kg)	− 64.95	38.93	− 141.2	11.35	0.100
	PMI	7.468	6.344	− 4.966	19.9	0.244
Spleen	(Constant)	345.1	134.1	–	–	–
	Maceration score 1 (ref = 0)	69.42	42.34	− 13.57	152.4	0.106
	*Maceration score 2 (ref = 0)*	*93.82*	*31.95*	*31.19*	*156.4*	***0.005***
	Gestational age (weeks)	− 0.814	5.127	− 10.86	9.234	0.874
	Gender (ref = female)	19.7	27.62	− 34.43	73.83	0.478
	*PM weight (kg)*	*− 57.09*	*25.62*	*− 107.3*	*−6.875*	***0.029***
	PMI	8.328	4.306	− 0.111	16.77	0.057
Right renal cortex	(Constant)	486.5	180.1	–	–	–
	Maceration score 1 (ref = 0)	111.6	57.66	− 1.452	224.6	0.057
	*Maceration score 2 (ref = 0)*	*153.5*	*43.24*	*68.72*	*238.2*	***0.001***
	Gestational age (weeks)	− 4.731	6.902	− 18.26	8.797	0.495
	Gender (ref = female)	31.01	37.31	− 42.12	104.1	0.409
	PM weight (kg)	− 22.15	34.8	− 90.36	46.06	0.526
	PMI	7.982	5.788	− 3.363	19.33	0.172
Liver	(Constant)	489.5	154.5	–	–	–
	*Maceration score 1 (ref = 0)*	*142.6*	*49.61*	*45.39*	*239.8*	***0.005***
	*Maceration score 2 (ref = 0)*	*209.9*	*37.25*	*136.9*	*282.9*	***< 0.001***
	Gestational age (weeks)	− 5.403	5.940	− 17.04	6.239	0.366
	Gender (ref = female)	− 41.84	32.13	− 104.8	21.13	0.197
	PM weight (kg)	1.241	29.93	− 57.43	59.91	0.967
	PMI	4.468	5.000	− 5.333	14.27	0.375
Right psoas	(Constant)	501.0	116.3	–	–	–
	*Maceration score 1 (ref = 0)*	*83.63*	*37.22*	*10.68*	*156.6*	***0.028***
	*Maceration score 2 (ref = 0)*	*161.3*	*27.91*	*106.6*	*216.0*	***< 0.001***
	Gestational age (weeks)	1.734	4.469	− 7.025	10.49	0.699
	*Gender (ref = female)*	*48.53*	*24.10*	*1.295*	*95.77*	***0.048***
	PM weight (kg)	− 35.18	22.56	− 79.39	9.034	0.123
	PMI	− 0.187	3.741	− 7.520	7.146	0.960

### Non-macerated perinatal deaths

In order to assess the relative contribution of maceration, we identified 14 neonatal deaths (7 males; 50%) which by definition had not undergone maceration. This sub-group had median PMI of 5 days (range 1–12 days) with median PM weight of 2.625 kg (range 0.339–4.335 kg). PMI was not a significant predictor of ADC values in any body area in this subsample.

### Reproducibility

Readers had the highest agreement in the right psoas muscle, with mean ratio of 1.04 and 95% limits of agreement (LOA) of the ratios between 0.80 and 1.35. Agreement was poorest in the spleen, with mean ratio of 1.02 and 95% LOA between 0.42 and 2.44. The mean ratio of agreement in all body areas was close to 1 (range between 0.95 and 1.10).

## Discussion

This study found that the maceration score but not PMI was significantly associated with post-mortem perinatal ADC body organ values. Caution should therefore be exercised when interpreting DWI PMMR to avoid attributing ADC changes to post-mortem interval.

The association of maceration with ADC values is supported in part by a previous study assessing post-mortem foetal brain ADC values, where this played a role in all regions of the brain analysed. Maceration is usually assessed at autopsy by external appearances, including skin changes, umbilical cord discoloration and cranial collapse, but the underlying cellular process remains poorly understood [10]. Changes in membrane permeability, redistribution of magnetization between compartments and increased diffusivity within the remaining extracellular space may all account for the diffusivity changes observed in our study [13] giving differences in ADC values. Clearly, a combination of autolysis and maceration contribute to the changes seen in this study.

When we analysed a subset of non-macerated foetuses, we did not find an underlying effect of post-mortem interval on ADC changes. Whilst previous studies in perinatal deaths have not been performed, a recently published study of 19 adult post-mortem cases [14] found that the ADC values of the liver did not significantly change with time (in hours) since death, with temperature changes accounting for a greater effect. Nevertheless, the ADC values in this study were measured at two hourly intervals over 16 h, and this may not have been enough time for effects to be observed.

Previous work performed for a set of 15 foetuses and neonates reported that ADC changes in the lung may be correlated to PMI [12]; however, in our larger cohort, we could not reproduce those findings. Of the 15 subjects that were analysed in the comparator study, the majority (*n* = 10) were estimated to have undergone in-utero retention ranging from between 0.5 and 2 days prior to delivery, with 4 subjects allocated a maceration score of ≥ 1 at autopsy. Due to the small sample size, neonatal deaths were not compared to intra-uterine deaths and the reported changes in lung ADC values with time may have therefore been a property of underlying maceration and autolysis rather than post-mortem interval.

Although we have assessed the use of ADC values in the largest perinatal population studied to date, our study has several limitations, with relatively small sample size and only fair inter-reader reproducibility. We speculate that this may be due to inter-observer variation in ROI creation, and limited experience in analysing post-mortem DWI imaging. We also recognise that the use of size-specific ROIs may have generated further variation, but a fixed size ROI would not have yielded accurate results across the range of foetal weights and gestations in our sample size. There may have been some restrictions in our technique, as all of our cases were imaged at 1.5 T and not 3 T. A higher magnetic field strength could have yielded greater image contrast for the anatomical referencing on isovolumetric T2 sequences to aid placement of body organ ROIs [15]; however, the accuracy of ADC values on high-field-strength MRI in the post-mortem setting has yet to be evaluated, and may be more susceptible to field inhomogeneity and artefacts [16]. In addition, whilst we used standard *b* values at 0, 500 and 1000 s/mm^2^, these may not be optimal for post-mortem imaging at low temperatures and truly quantitative imaging across this range could be useful in this setting. Nevertheless, our imaging techniques were standardised across all our subjects in this study following the same MRI protocol. We did not apply temperature correction to our data as the correlations and multivariate analysis would have been unaffected and we were not comparing our data with live subjects.

In addition, the usage of a maceration score is subjective, and higher scores do not always correlate with longer intra-uterine retention times [17]. Ideally, investigating the true effect of maceration would require knowing precisely the intra-uterine retention period. This is challenging in most intra-uterine deaths where the exact time of death is unknown (Fig. 1), but this may be possible following feticide for termination of pregnancy which could be assessed in future work. Equally, a larger subset of non-macerated cases could be investigated in more detail. It is clear that the cellular changes that are detected by DWI are complex and related to several factors, and thus we urge caution if considering using these features in medicolegal and forensic cases.

## Conclusion

The degree of maceration plays an important role when interpreting the post-mortem body organ ADC values, but we did not find a significant correlation with PMI even in cases where maceration was excluded. Further research is needed to understand the organ-specific changes during maceration for potential timing of intra-uterine demise in cases of stillbirth.

## Abbreviations

ADC: Apparent diffusion coefficient
DWI: Diffusion-weighted (magnetic resonance) imaging
(PM)MR: (Post-mortem) magnetic resonance imaging
PMI: Post-mortem interval
ROI: Region of interest
